# Comment on Altaf et al. Non-Thermal Plasma Reduction of Ag^+^ Ions into Silver Nanoparticles in Open Atmosphere under Statistically Optimized Conditions for Biological and Photocatalytic Applications. *Materials* 2022, *15*, 3826

**DOI:** 10.3390/ma17081750

**Published:** 2024-04-11

**Authors:** Dave Mangindaan

**Affiliations:** 1 Civil Engineering Department, Faculty of Engineering, Bina Nusantara University, Jl. KH. Syahdan 9, West Jakarta 11480, Indonesia; dave.mangindaan@binus.ac.id; 2 Waste-Food-Environmental Nexus Research Interest Group, Bina Nusantara University, Jl. KH. Syahdan 9, West Jakarta 11480, Indonesia

**Keywords:** plasma, AgNPs, silver nanoparticles, Box–Behnken, response surface methodology

## Abstract

Altaf et al. recently published in *Materials*, 2022; 15(11), 3826, about the synthesis of silver nanoparticles (AgNPs) using the non-thermal plasma reduction of AgNO_3_ salt and performed statistical optimization for the reaction conditions, i.e., (A) the concentration of a stabilizing agent, mM (B) concentration of AgNO_3_ salt, mM and (C) the reaction time, mins. We would like to point out that their writing on the statistical analysis (Box–Behnken response surface methodology for predicting the size of the nanoparticles) is not complete and, therefore, cannot be independently checked by the readers. The problems found in their report are as follows: the hard-to-find actual value of the uncoded units; a dubious claim about the middle levels of variable B (salt concentration); inconsistency in using coded vs. uncoded units in the table vs. the regression equation; and three center points with identical conditions give a dissimilar prediction of results. These serious issues need to be clarified and revised, as well as several writing errors, in order to uphold the standard of scientific publications.

## 1. Introduction

We are writing in response to the article by Altaf et al., *Materials*, 2022; 15(11), 3826, “Non-Thermal Plasma Reduction of Ag^+^ Ions into Silver Nanoparticles in Open Atmosphere under Statistically Optimized Conditions for Biological and Photocatalytic Applications” [1]. This article is in line with our experience of plasma treatment for material modifications [2,3,4,5]. In that article, silver nanoparticles (AgNPs) were prepared using the non-thermal plasma reduction of AgNO_3_ salt and a stabilizing agent (glucose). They also conducted statistical optimization (the Box–Behnken response surface methodology) for the reaction conditions, i.e., (A) the concentration of the stabilizing agent, mM, (B) the concentration of AgNO_3_ salt, mM, and (C) the reaction time, mins. We would like to highlight that their calculation for the statistical analysis for predicting the size of the AgNPs is questionable and, therefore, cannot be independently checked by the readers of this particular paper. The experimental conditions and the results (AgNPs size) in question can be observed in Table 1.

The report by Altaf et al. on the experimental parameters (as shown in Table 1) was written only as independent variables in the form of coded units (−1, 0, +1) for the Box–Behnken response surface methodology comprising 15 runs, where three of them are identical center points (A = 0, B = 0, and C = 0, middle levels, for runs 3, 9, and 14). These coded units were not accompanied by the actual or the uncoded units in the Materials and Methods section. They finally can be found, although indirectly, from the x- and y-axes labels of Figure 4a–c (page 9) [1], where, on one hand, A = 1–5 mM, B = 1–5 mM, and C = 30–60 min, or in other words, the middle levels for A must be 3 mM, for B = 3 mM, and for C = 45 min. This inconvenience must be corrected. On the other hand, the middle levels of A and B were stated to be 3 and 5 mM (page 10, lines 1–2 from the top). Therefore, these comparisons indicate that the middle level for B was not calculated correctly by Altaf et al.

These aforementioned issues result in more confusion when the regression equation is discussed. The authors used the second-order polynomial model (Equation (1)) for their model (Equation (2)), as shown below:

Y = β_0_ + β_1_A + β_2_B + β_3_C + β_12_AB + β_13_AC + β_23_BC + β_11_A^2^ + β_22_B^2^ + β_33_C^2^ (1)

Y_(AgNPs size)_ = 230.981 − 50.208A − 1.00097 B + 2.895 C − 0.042 AB − 0.79 AC − 0.00029 BC + 3.549 A^2^ + 0.0201 B^2^ + 0.224 C^2^ (2)

When we checked Equations (1) and (2) against the center points (or with the conditions of A = B = C = 0, as shown in Table 1), then A = B = C = AB = AC = BC = A2 = B2 = C2 = 0. Therefore, the equations must yield Y = β_0_ or Y(AgNPs size) = 230.981 nm. However, in Table 1, the laboratory results of the AgNPs’ size did not exceed 40 nm, let alone 230 nm. Therefore, we expect that Equation (2) might be using uncoded units. However, with the aforementioned discrepancies of coded and uncoded units, the regression model in Equation (2) cannot be checked and verified. This issue must be corrected soon.

Finally, as highlighted in yellow in Table 1, the center points (A = B = C = 0, runs 3, 9, and 14) show different laboratory results (with AgNP sizes of 36.45, 32.6, and 25.55 nm, respectively). By performing a quick calculation, the average of these three values obtained from the laboratory is 31.5333 nm, with a standard deviation of 5.527 nm. This kind of broad inconsistency could somehow be tolerated for the laboratory-based experimental results. However, Altaf and coworkers’ prediction using the Box–Behnken regression calculation for the three identical center points (runs 3, 9, and 14) does not provide identical AgNP dimensions, i.e., 37, 32.3, and 22.36 nm, respectively (or an average of 30.5333 nm, and standard deviation of 7.477 nm). On the contrary, our prediction (as shown in Equation (3)) for the three identical center points shows that the predicted size of AgNPs is, in fact, 31.5333 nm (matching the average laboratory-based value), with a standard deviation of 0.0 nm. This discrepancy must be rectified.

Y(coded values) = 31.5333 − 2.16375A − 0.56875B − 3.7825C − 1.5575AB −0.73 AC −0.385BC − 3.03542 A^2^ − 3.39042 B^2^ − 1.80292 C^2^ (3)

**Figure 1:**
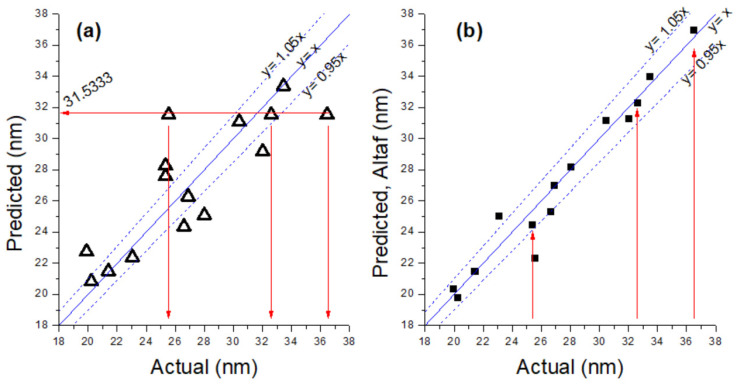
Parity plot of actual values against predicted values, guided with y = x, y = 0.95x, and y = 1.05x, representing a matching level of 100%, 95%, and 105%, respectively, for (**a**) our parity plot (△), and (**b**) that of Altaf and coworkers (■).

To show how realistic our regression model is vs. Altaf and coworkers’, we made a parity plot, as shown in Figure 1. Our parity plot in Figure 1a shows the different AgNP sizes recorded in Table 1 with runs 3, 9, and 14 (center points, with A = B = C = 0) modeled to one value of 31.5333 nm. On the other hand, the parity plot for the work by Altaf et al. (Figure 4b) shows that different values of the center points (runs 3, 9, and 14) are not modeled to a single value. This is very unrealistic, especially when the regression model falsely claims that it matches almost all the predicted values within the range of a ±5% error.

Besides this critical statistical problem, there are other issues based on the comments from a reviewer with high attention to detail to the original manuscript by Altaf and coworkers [1]. In Table 4 (page 14) [1], the difference in the size of the particles is very large, where those measured using XRD are 19–33 nm, while those measured via SEM are 82–102 nm. Moreover, the UV-vis peak of the AgNPs (20–80 nm) purchased by the reviewer has a maximum peak of UV-vis spectroscopy from 395 (AgNPs 20 nm) to 466 nm (AgNPs 80 nm). However, in Altaf and coworkers’ paper (Figure 4 and page 12), for the AgNPsat19.8 nm, the claimed peak is 468 nm, which leads to some uncertainty. In addition to those aforementioned issues, typographical errors are also found and shown in Table 2. These errors are essential to be revised.

## 2. Conclusions

In this note, we point out these serious issues that need to be clarified and revised heavily for the statistical parts, as well as several writing errors, in order to uphold the standard of scientific publications.

## Figures and Tables

**Table 1:** Experimental conditions, the AgNPs’ size (laboratory data), the prediction of AgNPs’ size (Altaf and coworkers), and our prediction accompanied by a step-by-step calculation.

					β_0_ = 31.5333	β_1_ = −2.16375	β_2_ = −0.56875	β_3_ = −3.7825	β_12_= −1.5575	β_13_ = 0.73	β_23_ = 0.385	β_11_ = −3.03542	β_22_ = −3.39042	β_3_ = −1.80292	Summation	
No.	A (Stabilizing Agent, mM)	B (AgNO_3_Salt, mM)	C (Reaction Time, mins)	AgNPs’Size (nm, Laboratory Data)		Multiplication of β with A	Multiplication of β with B	Multiplication of β with C	Multiplication of β with AB	Multiplication of β with AC	Multiplication of β with BC	Multiplication of β with A^2^	Multiplication of β with B^2^	Multiplication of β with C^2^	Our Prediction (nm)	Altaf et al.’s Prediction (nm)
1	0	1	1	23.05	31.5333	0	−0.56875	−3.7825	0	0	0.385	0	−3.39042	−1.80292	22.37	25.07
2	1	0	1	21.4	31.5333	−2.16375	0	−3.7825	0	0.73	0	−3.03542	0	−1.80292	21.48	21.5
3	0	0	0	36.45	31.5333	0	0	0	0	0	0	0	0	0	31.53	37
4	0	−1	1	19.89	31.5333	0	0.56875	−3.7825	0	0	−0.385	0	−3.39042	−1.80292	22.74	20.38
5	−1	0	1	26.6	31.5333	2.16375	0	−3.7825	0	−0.73	0	−3.03542	0	−1.80292	24.35	25.32
6	−1	0	−1	33.45	31.5333	2.16375	0	3.7825	0	0.73	0	−3.03542	0	−1.80292	33.37	34
7	−1	1	0	25.33	31.5333	2.16375	−0.56875	0	1.5575	0	0	−3.03542	−3.39042	0	28.26	24.5
8	1	−1	0	28	31.5333	−2.16375	0.56875	0	1.5575	0	0	−3.03542	−3.39042	0	25.07	28.2
9	0	0	0	32.6	31.5333	0	0	0	0	0	0	0	0	0	31.53	32.3
10	−1	−1	0	26.88	31.5333	2.16375	0.56875	0	−1.5575	0	0	−3.03542	−3.39042	0	26.28	27
11	0	−1	−1	30.4	31.5333	0	0.56875	3.7825	0	0	0.385	0	−3.39042	−1.80292	31.08	31.2
12	1	1	0	20.22	31.5333	−2.16375	−0.56875	0	−1.5575	0	0	−3.03542	−3.39042	0	20.82	19.8
13	1	0	−1	25.33	31.5333	−2.16375	0	3.7825	0	−0.73	0	−3.03542	0	−1.80292	27.58	24.5
14	0	0	0	25.55	31.5333	0	0	0	0	0	0	0	0	0	31.53	22.36
15	0	1	−1	32.02	31.5333	0	−0.56875	3.7825	0	0	−0.385	0	−3.39042	−1.80292	29.17	31.33

**Table 2:** Typographical errors found in Altaf et al [1].

No.	Location in [1]	Type of Errors	Corrections to Be Performed
1.	Reference 6	Scientific names must be written in italics	*Cestrum nocturnum*
2.	Reference 8	Scientific names must be written in italics	*Lactuca sativa*
3.	Reference 10	Scientific names must be written in italics	*Jasminum subtriplinerve*
4.	Reference 14	Chemicals must be written with correct subscripted numbers	Ag–SiO_2_
5.	Reference 15	Scientific names must be written in italics	*Ziziphus jujuba*
6.	Reference 21	Scientific names must be written in italics	*Aspergillus terreus*
7.	Reference 29	Scientific names must be written in italics	*Pulicariaglutinosa*
8.	Reference 39	Ions must be written with appropriate superscripted numbers	Ag^+^ to Ag^0^
9.	Reference 41	Scientific names must be written in italics	*Ficus retusa*
10.	Reference 54	Scientific names must be written in italics	*Cuscuta japonica*
11.	Reference 57	Scientific names must be written in italics	*Catharanthus roseus*
12.	Reference 61	Chemicals must be written with correct subscripted numbers	ZnFe_2_O_4_
13.	Reference 62	Scientific names must be written in italics	*Sambucus ebulus*
14.	Page 2	Ions must be written with appropriate superscripted numbers	..DC plasma…convert Ag^+^ ions…
15.	Section 2.3 paragraph 1, Section 2.3 paragraph 2, Section 3.1	The word “2nd” must be written with superscripted nd.	..into a 2nd order polynomial… ..2nd order interaction… ..a 2nd order polynomial…
